# A Surgeon's Challenge: Diagnosing and Managing Hidden Bile Duct Stones Post-cholecystectomy

**DOI:** 10.7759/cureus.56865

**Published:** 2024-03-25

**Authors:** Guangbin Chen, Ke Wang, Yanguang Sha, Dingbang Wang, Zhigang Liu

**Affiliations:** 1 Department of Hepatobiliary Surgery, The Second People's Hospital of Wuhu, Wuhu Hospital Affiliated to East China Normal University, Wuhu, CHN; 2 Graduate School, Wannan Medical College, Wuhu, CHN

**Keywords:** multidisciplinary management, advanced imaging, post-cholecystectomy complications, laparoscopic cholecystectomy, choledocholithiasis

## Abstract

This case report details the diagnostic and management challenges encountered with hidden bile duct stones post-cholecystectomy in a 58-year-old female patient. Despite a successful laparoscopic cholecystectomy, the patient developed sudden upper abdominal pain and jaundice, leading to the discovery of an impacted bile duct stone. The case underscores the limitations of conventional preoperative diagnostics and highlights the importance of advanced imaging techniques and a multidisciplinary approach for optimal outcomes. The successful extraction of the stone via endoscopic retrograde cholangiopancreatography (ERCP) with sphincterotomy demonstrates the efficacy of this therapeutic strategy. This report emphasizes the need for heightened vigilance and comprehensive evaluation in the postoperative management of gallstone disease, contributing valuable insights into the complexities of choledocholithiasis post-cholecystectomy.

## Introduction

In the realm of gallstone disease management, the post-cholecystectomy period poses unique challenges, particularly with the occurrence of hidden bile duct stones [1]. Despite the widespread adoption of laparoscopic cholecystectomy (LC) for its minimally invasive benefits, patients can still face significant postoperative complications, such as choledocholithiasis [2]. This condition not only complicates recovery but also necessitates a nuanced approach to diagnosis and treatment. Traditional diagnostic tools often prove inadequate for identifying these concealed stones, highlighting the need for advanced imaging techniques and a collaborative, multidisciplinary strategy for effective management [2,3]. This case report delves into the complexities of diagnosing and managing hidden bile duct stones post-cholecystectomy, underscoring the critical need for vigilance and comprehensive evaluation in achieving optimal patient outcomes.

## Case presentation

A 58-year-old female patient was admitted to the hospital due to recurrent discomfort and bloating pain in the right upper abdomen for the past six months. The symptoms intensified with physical activity and after meals but subsided upon resting. The patient did not exhibit symptoms such as chills, high fever, nausea, or vomiting. Upon evaluation in our department, no significant signs of jaundice were observed in the skin or sclera. The patient's appetite, bowel movements, sleep, and mental state remained unaffected. Her medical history included hypertension and type II diabetes, which she managed for five years, and a prior surgical intervention for a left humerus fracture.

**Figure 1:**
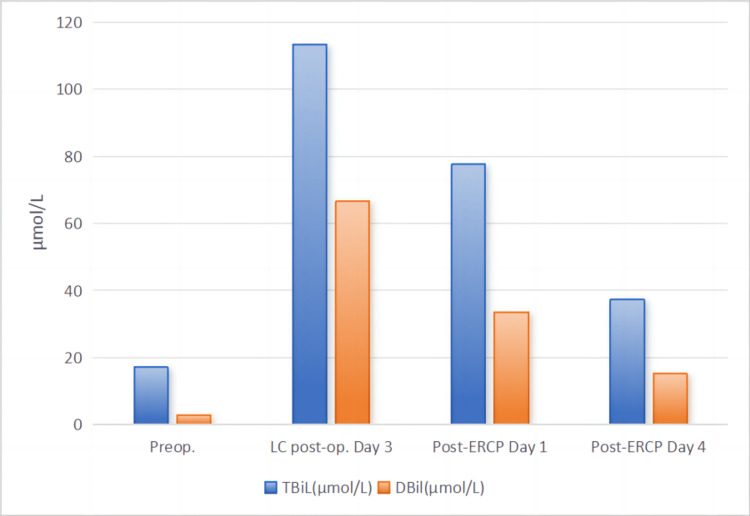
Perioperative changes in bilirubin. Preop: preoperative; LC: laparoscopic cholecystectomy; op: operative; ERCP: endoscopic retrograde cholangiopancreatography; TBiL: total bilirubin; DBil: direct bilirubin

Diagnostic tests conducted upon admission revealed the following serological indicators: white blood cells (WBC) at 6.68×10^9^/L, neutrophil percentage (NEUT%) at 49.5%, and liver function tests indicating alanine aminotransferase (ALT) at 32 U/L, aspartate aminotransferase (AST) at 21 U/L, gamma-glutamyl transferase (γ-GT) at 24 U/L, alkaline phosphatase (ALP) at 108 U/L, total bilirubin (TBiL) at 17.1 μmol/L, direct bilirubin (DBil) at 2.8 μmol/L (Figure 1), and amylase (AMY) at 66 U/L. Tumor markers were within normal limits: alpha-fetoprotein (AFP) at 2.41 ng/ml, carcinoembryonic antigen (CEA) at 1.2 ng/ml, and carbohydrate antigen 19-9 (CA19-9) at 22 U/ml. An ultrasound examination revealed chronic cholecystitis, multiple gallstones with the largest measuring approximately 2.0×1.4 cm, and no significant dilation or abnormal echoes observed in the upper segment of the common bile duct.

**Figure 2:**
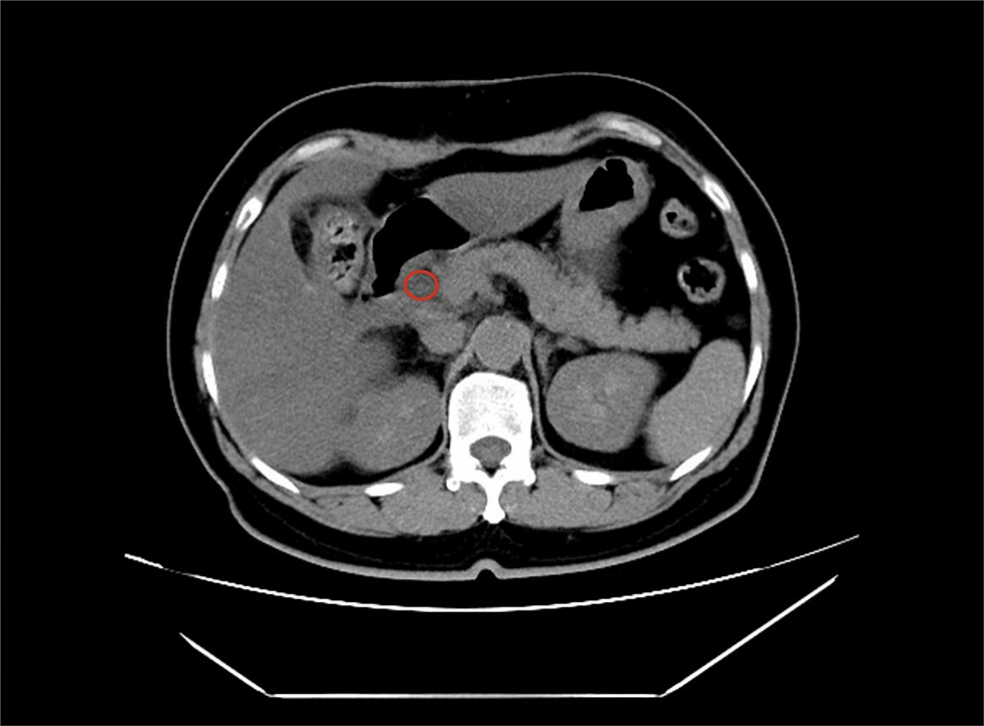
Postoperative CT scan of the upper abdomen showed a stone (marked in red) in the lower part of the common bile duct. CT: computed tomography

**Figure 3:**
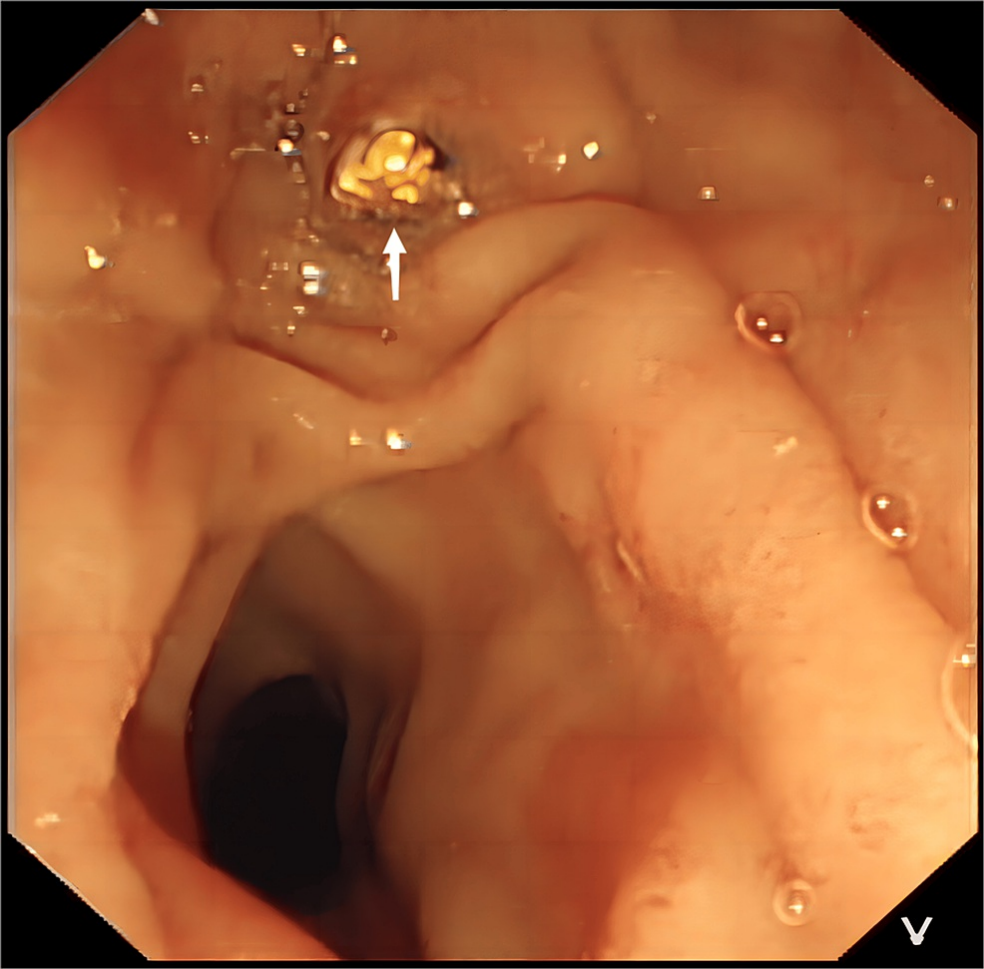
Postoperative ERCP examination suggests an impacted stone (white arrow) at the duodenal papilla. ERCP: endoscopic retrograde cholangiopancreatography

The patient underwent a routine LC on November 17, 2023, which was executed successfully. However, on the first postoperative day, she developed sudden upper abdominal pain and noticeable jaundice in the sclera and skin. A subsequent reevaluation of her liver function revealed significantly elevated levels: ALT at 615 U/L, AST at 541 U/L, γ-GT at 380 U/L, ALP at 325 U/L, TBiL at 113.3 μmol/L, and DBil at 44.0 μmol/L (Figure 1), with AMY at 516 U/L. An emergency abdominal CT scan suggested a common bile duct stone (Figure 2). Further investigation via endoscopic retrograde cholangiopancreatography (ERCP) identified an impacted stone at the duodenal papilla (Figure 3). An endoscopic sphincterotomy (EST) was performed, and the stone was successfully extracted using a basket. Following the procedure, the patient's symptoms were alleviated, and her liver function normalized (Figure 1).

## Discussion

The management of choledocholithiasis, particularly when it presents after LC, poses a significant challenge to surgeons worldwide [4,5]. This case report underscores the complexities involved in diagnosing and treating hidden bile duct stones post-cholecystectomy, a scenario that is not uncommon but often underreported in clinical literature.

In recent years, the global incidence of gallbladder stones has seen a notable increase, attributed largely to dietary changes and improvements in quality of life [1,6,7]. Concurrently, the prevalence of choledocholithiasis, especially in the postoperative phase, has become a point of concern [1]. Our case highlights a patient who, following a seemingly successful LC, presented with symptoms indicative of bile duct obstruction. This situation underscores the critical need for a high index of suspicion for choledocholithiasis in patients post-cholecystectomy, especially when presenting with jaundice, abdominal pain, and elevated liver enzymes.

In most countries and regions, routine preoperative evaluations for cholecystectomy typically include liver function tests and abdominal ultrasound examinations [7-9]. If these indicators do not reveal abnormalities, further investigations, such as magnetic resonance cholangiopancreatography (MRCP), are often not pursued, particularly in cost-sensitive healthcare settings. However, as demonstrated in our case, these methods may not always be sufficient. The stone in question, measuring approximately 0.8 cm in diameter, was not detected by ultrasound examination preoperatively due to its floating nature in the lower segment of the common bile duct, coinciding with normal liver function tests at the time. This oversight brings to light the limitations of conventional diagnostic tools and the potential role of more advanced imaging techniques, such as MRCP, in the preoperative evaluation of gallstone disease.

The therapeutic strategies for managing choledocholithiasis post-LC include ERCP with sphincterotomy and stone extraction [9,10], which was successfully employed in our case. This method, while effective, is not without risks and underscores the importance of a multidisciplinary approach in the management of these patients. The decision-making process should involve a thorough evaluation of the risks and benefits, patient preferences, and the expertise available within the treating institution.

## Conclusions

This case report serves as a critical reminder of the challenges and complexities in diagnosing and managing hidden bile duct stones post-cholecystectomy. It underscores the need for vigilance, the potential limitations of preoperative diagnostics, and the importance of considering advanced imaging techniques in the preoperative assessment of patients with gallstone disease. Moreover, it highlights the significance of a multidisciplinary approach in ensuring optimal outcomes for patients facing this complex condition.
